# Correction: Cross border project in China-Pakistan economic corridor and its influence on women empowerment perspectives

**DOI:** 10.1371/journal.pone.0303621

**Published:** 2024-05-08

**Authors:** The PLOS ONE Editors

## Notice of Republication

This article was republished on April 24, 2024, to address a file issue. An updated version of S1 File is provided with this notice. Please download this article again to view the correct version.

## Supporting information

S1 File Survey results. (XLSX)
